# Fermentation optimization and disease suppression ability of a *Streptomyces* ma*. FS-4* from banana rhizosphere soil

**DOI:** 10.1186/s12866-019-1688-z

**Published:** 2020-01-31

**Authors:** Yajie Duan, Jian Chen, Wei He, Jingjing Chen, Zhencai Pang, Huigang Hu, Jianghui Xie

**Affiliations:** 1 Key Laboratory of Tropical Fruit Biology, Ministry of Agriculture, Chinese Academy of Tropical Agricultural Science, South Subtropical Crop Research Institute, Zhanjiang, 524091 China; 2 College of Food Science and Technology, Hainan University, Haikou, 570228 China

**Keywords:** *Streptomyces manipurensis*, Fusarium wilt of banana, *FS-4*, Antimicrobial activity, Culturing conditions

## Abstract

**Background:**

*Fusarium* wilt of banana is one of the most destructive diseases in banana-growing regions worldwide. Soil-borne diseases and soil microbial communities are closely related. The screening of antagonistic bacteria from soil microorganisms in areas with *Fusarium* wilt of banana is of great practical significance for controlling this disease.

**Results:**

A strain designated *FS-4* was isolated from healthy banana rhizosphere soil in an area affected by *Fusarium* wilt. This strain exhibited a significant antagonistic effect on the pathogen. Pot experiments revealed that the fermentation broth of strain *FS-4* not only decreased the incidence of banana Fusarium wilt, but also promoted the growth of banana seedlings. The strain was identified as *Streptomyces* ma. by its morphological, physiological, and biochemical characteristics and 16S rRNA gene sequence analysis. The culture and fermentation conditions for this strain were optimized by single-factor and response surface experiments. The optimum culture conditions for *Streptomyces* ma*. FS-4* were as follows: peptone 0.5%, saccharose 2.4, 0.05% K_2_HPO_4_, 0.05% MgCl_2_, and 0.05% NaCl at an initial pH of 7.0; 180 g at 28 °C; and inoculation size of 6% for 62 h. The diameter of bacteriostasis circle for *Bacillus subtilis* reached 26.7 mm.

**Conclusion:**

*Streptomyces* ma*. FS-4* is an important microbial resource as a biological agent for the control of plant pathogenic fungi and can be used to promote banana growth.

## Background

*Fusarium* wilt of banana is a soil-borne disease caused by fungal infection (*Fusarium oxysporum* f. sp. *cubense*, *Foc*) of the banana root [1–3]. Since the 1990s, *Fusarium* wilt of banana has spread to the world’s leading banana production areas and has become one of the most destructive banana diseases [4–6]. In recent years, *Fusarium* wilt of banana has spread in major areas of China, thereby seriously endangering the healthy and sustainable development of the banana industry [2, 5–7]. *Fusarium* wilt of banana is extremely infectious and difficult to cure. The pathogen has strong resistance to stress and can survive for 30 years in soil, thereby causing extensive destruction of orchards [4, 8, 9].

Actinomycetes are widely distributed in natural ecosystems. They have various metabolic functions and are widely used as a biological resources [10–14]. Actinomycetes have long been an important source of bioactive metabolites. More than half of existing natural antibiotics are produced from actinomycetes (especially *Streptomyces*) [15–17]. However, among the antibiotics produced from actinomycetes, only extremely small doses will exert a significant effect and can effectively prevent and control a variety of infectious diseases in humans and livestock [16, 18, 19]. For agricultural disease control, researchers have screened different actinomycete strains from soils and plants. These strains can control turfgrass root rot [20], rice blast [21], rice leaf blight [18], and tomato gray mold [22] with good results. Moreover, the antimicrobial active substances in the metabolites of antagonistic actinomycetes are preliminarily isolated [17, 23, 24]. Roy et al. (2006) isolated dibutyl phthalate from *Streptomyces* fermentation liquid; this compound has a strong inhibitory effect on G^+^ and G^−^ bacteria and unicellular and filamentous fungi. Mingma et al. (2014) isolated actinomycetes from legumes fermentation liquid and identified phenylacetic acid as the active substance that inhibited the pathogen of soybean glycine *Xanthomonas* [14]. These results provided new ideas and approaches for the biological control of *Fusarium* wilt of banana.

The production level of microbial fermentation not only depends on the performance of the production strains, but also needs the best environmental conditions, i.e., the fermentation process, to fully display its production capacity [5]. Therefore, it is necessary to study the optimum fermentation process conditions for strain production, such as nutritional requirements, culture temperature, pH conditions, and time; accordingly, a reasonable fermentation process is designed for the production of strains under the best product synthesis conditions, thereby achieving high quality and yield [1].

In our study, an actinomycete strain with strong antagonistic effect on *Fusarium* wilt was identified from rhizosphere soil with severe banana disease, and its species relationship and fermentation conditions were studied. Our results provide an experimental basis for the separation and identification of antibiotics and the theoretical basis for their production and application.

## Methods

### Sampling site and sample collection

Twenty-five soil samples were collected from healthy banana rhizosphere soil in an area affected with *Fusarium* wilt of banana in Zhanjiang City, Guangdong Province in November of 2015. From a healthy plantain garden where bananas has been grown for more than 10 years, rhizosphere soil was collected at 10–30 cm of the root of healthy plants. Soil samples were collected and placed inside a sterile plastic bag, sealed, and preserved in an ice box. In the laboratory, the roots and stones were removed and stored at 4 °C before the actinomycetes were isolated.

Pathogenic fungi strains includes *Fusarium oxysporum* f. sp. *cubense Race1* (FOC.1), *F. oxysporum* f. sp. *cubense Race* 4 (FOC.4), *Curvulatia fallaxis* (CFO), *Colletotrichum gloeosporides* (CG), and *Alternaria tenuissima Maa* (MAA), *Escherichia coli* (*E. coli*), *Bacillus subtilis* (*B. subtilis*), and *Staphylococcus aureus* (*S. aureus*)were provided by the Institute of Environment and Plant Protection. Taq PCR Master Mix and bacterial genomic DNA rapid extraction kit were purchased from Sigma-Aldrich Co. (USA).

“Gause’s no. 1 culture”: 20.0 g Soluble starch, 0.5 g NaCl, 1.0 g KNO_3_, 0.5 G K_2_HPO _4_·3H_2_O, 0. 5 g MgSO _4_·7H_2_O, 0.01 g FeSO _4_·7H_2_O, 20 g agar, 1000 mL H_2_O

### Isolation and purification of actinomycetes

Actinomycetes were isolated by serial dilution method on Gause’s no. 1 culture [13, 19, 21, 24, 25], which was sterilized at 121 °C for 20 min, static, and cooled to room temperature. In total, 3 mL of 1% dichromate solution after filter sterilizer was added to the culture (per liter); 0.5 mL of 5% mycetin was set aside for reverse plate. Soil sample at 10 g was dissolved in 100 mL aseptic water. The suspension was mixed well and then diluted 10 times to prepare 10^− 4^, 10^− 5^, and 10^− 6^ suspension. Next, 0.1 mL of suspension was added to the Gause’s no. 1 culture. The gradient was set to three iterations and incubated at 28 °C for 4 days. After a single colony was purified, it was transferred to Gause’s no. 1 culture for 4 days [14, 15, 26]. The serial number was preserved.

### Screening of antagonistic Actinomycetes

The antimicrobial activity of the isolated actinomycetes was screened by double-layer agar method. After culturing for 5 days, actinomycetes were cut into pieces with a perforator (Φ = 8 mm) and moved to the center of Gause’s no. 1 culture. They were then grown at 28 °C for 2 days for colonization. *Fusarium* wilt of banana fungal pathogen was prepared for the fungi suspension (6 × 10^8^ CFU/mL) with aseptic water and evenly sprayed on the plate with a 2-day culture of actinomycetes. Suspension was poured into 15 mL Nutrient Agar (NA) culture (the formula: 10.0 g protein, 3.0 g beef powder, 5.0 g sodium chloride and 15.0 g agar were dissolved in 1 L distilled water, pH was adjusted to 7.3) at 40 °C. Each treatment was repeated thrice. A plate without actinomycetes was used as the control. The diameter of the inhibition circle was measured at 28 °C for 24 h [16, 23, 25–28].

### Inhibitory effect of antagonistic Actinomycetes on the growth of pathogenic Fungi and Bacteria

Before the inhibitory experiment, the screened antagonistic actinomycete was cultured for 5 days, and pathogenic fungal cake was prepared by sterile culture (Φ = 8 mm). The fungi were placed at the center of a PDA flat [14, 15]. After culturing for 2 days at 28 °C, five kinds of pathogenic fungal cakes were inoculated symmetrically 2 cm apart from both sides of the actinomycetes by using the same method. A fungal disc was placed in the center of the plate. A fungal mycelia disc alone in the center of the plate served as a control. The diameters of the inhibition zones were measured after incubation for 7 days at 28 °C. Each treatment was repeated thrice, and the width of the inhibition band was utilized to measure the inhibitory effect of this strain on pathogenic fungi. *E. coli*, *S. aureus*, and *B. subtilis*, were made into bacterial suspension (1 × 10^8^ cfu/ml) with sterile water. The paper soaked with actinomycetes was evenly placed in a 9 cm plate coated with 200 mL bacterial solution at 37 °C and cultured for 24 h. The diameter of the bacteriostat was measured. Meanwhile, paper soaked in normal saline was used as the control. We aimed to analyze the inhibitory effect of the screened antagonistic actinomycete on pathogens and bacteria.

### Pot culture experiments

Pot culture experiments were performed under greenhouse conditions, i.e., 28 °C, 70% humidity, and sufficient natural light. The fermentation broth of actinomycete *FS-4* was inoculated in sterilized Gause’s no. 1 culture and incubated with shaking (150 rpm) at 28 °C for 7 days. The fermentation broth was filtered then diluted 50-fold, and 50 ml of the liquid was inoculated into the banana seedlings to establish four treatment groups, as follows: CK1 (non-inoculated FOC.4 and application of sterile water); CK2 (inoculated FOC.4 and application of sterile water); CK3 (inoculated FOC.4 and polymycin); and A (inoculated FOC.4 and fermentation broth of actinomycete *FS-4*). Banana seedlings with consistent growth rate of 5–6 leaves were selected and soaked in pathogen suspension at a concentration of 10^7^ cfu /mL. Then, they were transplanted into a plastic bowl containing 700 g soil and filled with 50 mL pathogen suspension at the rhizosphere soil of banana seedlings. The banana seedlings were cultivated in a greenhouse. Each experiment was repeated thrice. The watering treatment liquid A and the positive control CK3 were applied to the roots of the banana seedlings. The control treatment of the wilt disease was water treated with water for several days. The disease index and the disease prevention effect of banana seedlings transplanted during 49 days were calculated by the following formula. The average fresh biomass and incidence of banana seedlings were determined on the 49th day.

$$ \mathrm{Disease}\ \mathrm{index}=\frac{\sum \left(\mathrm{number}\ \mathrm{of}\ \mathrm{disease}\mathrm{d}\ \mathrm{plants}\times \mathrm{representative}\ \mathrm{value}\right)}{\mathrm{the}\ \mathrm{sum}\ \mathrm{of}\ \mathrm{the}\ \mathrm{number}\ \mathrm{of}\ \mathrm{plants}\times \mathrm{the}\ \mathrm{representative}\ \mathrm{value}\ \mathrm{of}\ \mathrm{the}\ \mathrm{most}\ \mathrm{serious}\ \mathrm{disease}}\times 100\% $$

$$ \mathrm{Disease}\ \mathrm{prevention}\ \mathrm{effect}\ \left(\%\right)=\frac{\mathrm{control}\ \mathrm{disease}\ \mathrm{index}-\mathrm{treatment}\ \mathrm{of}\ \mathrm{disease}\ \mathrm{index}\ }{\mathrm{control}\ \mathrm{disease}\ \mathrm{index}}\times 100\% $$

### Classification and identification of strains

#### Observation of morphological characteristics

Sterilized glass was inserted into the Gause’s no. 1 culture inoculated with actinomycetes at 45 °C. Coverslips were taken out after incubation at 28 °C for 21 days [14, 21]. Aerial hyphae, hypha, and spore characteristics were observed with a transmission electron microscope.

#### Physiological and biochemical characteristics

The target actinomycetes were cultured at 28 °C for 21 days. The color of hyphae and airborne hyphae and the existence of soluble pigments were observed [15, 21].

#### Phylogenetic characteristics

Genomic DNA of *FS-4* was isolated using the bacterial genomic DNA rapid extraction kit (Sigma-Aldrich Co., Ltd., USA) and the bacterial 16S rDNA was amplified using the universal primers 27F (5′-AGAGTTTGATCCTGGCTCAG-3′) and 1492R (5′-GGTTACCTTGTTACGACTT-3′). PCR amplifications were performed with a Trio PCR System (Biometra, Germany), in a total volume of 25 μl consisting of template DNA (2.0 μL), 2 × Taq PCR Master Mix (12.5 μl), ddH_2_O (8.5 μl), Upstream primer (1 μL) and Downstream primer (1 μL). The conditions for thermal cycling were as follows: denaturation of the DNA at 94 °C for 5 min; and 35 cycles at 94 °C for 30 s, primer annealing 30 s at 55 °C for template DNA, and DNA elongation at 72 °C for 90 s.

PCRs were performed in the Trio PCR System (Biometra, Germany). The PCR system and conditions were as described by Himaman et al. [29]. The PCR amplification products were visualized by 1.0% (w/v) agarose gel electrophoresis. The amplified PCR products were sequenced by a Sanger-based, automated sequencer (Applied Biosystems).

The product was purified and sequenced. GenBank was used to search for sequence similarity, and the sequence of the pattern strain with high similarity was selected. The homology was compared by using the neighbor-joining method in MEGA5.0. A phylogenetic tree was constructed to determine the taxonomic position of the actinomycetes [19, 22].

### Optimization of the conditions for improving the bacteriostatic activity of the Actinomycete *FS-4* fermentation broth

After cultivation in Gause’s no. 1 culture at 37 °C at 120 r/min, precipitation for 24 h at 4 °C, and autoclaving at 121 °C and 0.1 MPa, treatment with actinomycetes *FS-4* antagonistic bacterial fermentation broth was performed for 20 min.

#### Effects of Different Carbon and Nitrogen Sources

Under the same culture conditions, 2% glucose, saccharose, soluble starch, corn flour, and lactose were used to replace the soluble starch in the basic fermentation culture. Then, 0.1% of ammonium sulfate, monarkite, nitrate potash, yeast powder, and peptone instead of KNO_3_ were respectively added to the basic fermentation culture. The best carbon and nitrogen sources were determined thrice according to the method described in Section 2.4.

#### Effect of Inorganic Salt Content

The concentrations of sodium chloride, potassium hydrogen phosphate, and magnesium sulfate were set at 0.025, 0.050, 0.075, 0.100, and 0.125%. The best inorganic salt content was determined thrice according to the method described in the section on the screening of indicator strains.

#### Effects of Initial pH, Fermentation Temperature, and Time

The initial pH of the culture was set at 5–9, temperature was set at 22 °C–34 °C, and time was at 48–144 h. The optimum initial pH value, fermentation temperature, and time were determined thrice according to the method described in the section on the screening of indicator strains.

### Optimization of fermentation conditions by response surface analysis

On the basis of the single-factor experiment, sucrose, peptone, and fermentation time were selected as the investigation factors, and response surface analysis was carried out according to the method described in the section on the screening of indicator strains.

### Statistical analysis

Design-Expert V8.0.6 software was used to design the Box–Behnken experiment. All the experiments were repeated thrice, and ANOVA was carried out by SPSS 19.0.

## Results

### Isolation and screened of Actinomycetes

The strains isolated from soil samples were screened in accordance with the colony morphology and color similarity on the purified culture, and the corresponding actinomycetes were obtained. After preliminary screening by plate confrontation culture method and re-screening by the oxford cup method, one of the largest actinomycetes produced in the inhibition zone was identified as *FS-*4. By combining traditional taxonomy with 16S rRNA gene sequence, the preliminary identification of *Streptomyces* ma*. FS-4* achieved the maximum sequence similarity with *Streptomyces.* ma, reaching 99%. The phylogenetic tree created based on sequence indicated that the strains were in the same family.

### Antimicrobial activity evaluation of strain *FS-4*

The inhibitory effect of strain *FS-4* fermentation products on three kinds of bacterial was shown in Table 1. The fermentation products have no inhibitory effect on *S. aureus* and had better inhibitory effect on *B. subtilis* than *E. coli*. Therefore, *Bacillus subtilis* was selected as the indicator bacterium for the next study.

**Table 1:** Inhibitory effects of strain *FS-4* against three bacterial strains

Bacterial strain	*Escherichia coli*	*Bacillus subtilis*	*Staphylococcus aureus*
Bacteriostasis band width (mm)	12.5 ± 0.5	16.5 ± 0.8	–

Strain *FS-4* exerted some inhibitory effects on *Fusarium oxysporum* f. sp. *cubense Race1* (FOC.1), *F. oxysporum* f. sp. *cubense Race* 4 (FOC.4), *Curvulatia fallaxis* (CFO), *Colletotrichum gloeosporides* (CG), and *Alternaria tenuissima Maa* (MAA) (Table 2). The antagonism to CG was the strongest.

**Table 2:** Inhibitory effect of strain *FS-4* against five plant pathogenic fungal strains

Fungus	Bacteriostasis band width (mm)
FOC.1	10.07 ± 0.34b
FOC.4s	12.12 ± 0.56 a
CFO	12.26 ± 0.87 ab
MAA	9.11 ± 0.34b
CG	13.61 ± 0.79 bc

Note: Small-case letters represent 5% significant level

**Fig. 1:**
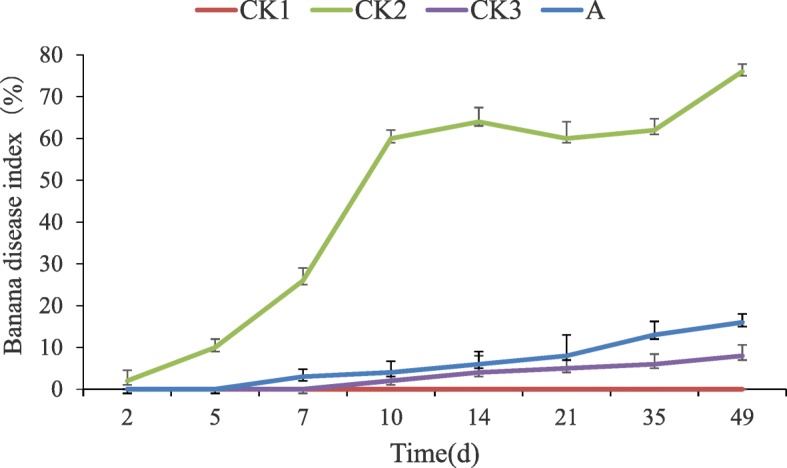
Effect of *Streptomyces* ma. *FS-4* on banana *Fusarium* wilt

**Fig. 2:**
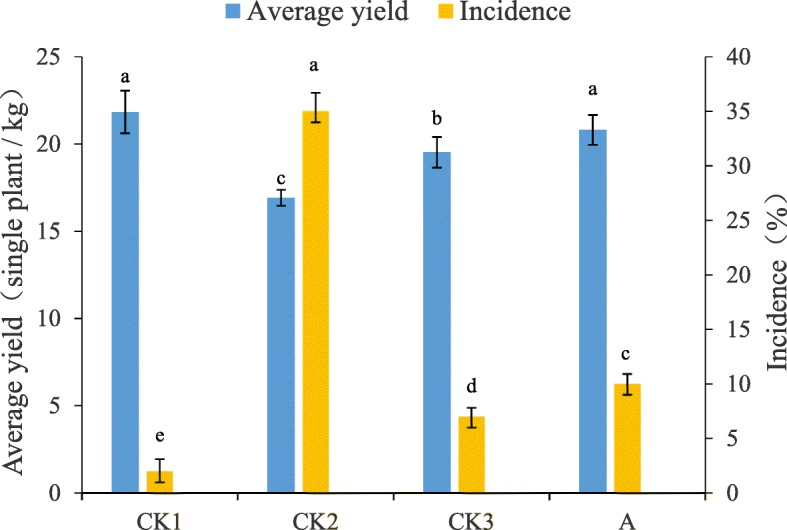
Effect of *Streptomyces* ma. *FS-4* on banana average yield and disease incidence. Note: Different lowercase letters show significant difference at *P* < 0.05

When the plants were inoculated with pathogen at a high concentration of FOC.4 (1 × 10^7^ cfu /mL) for 7 days, the blight rapidly spread. The disease began to appear in group A at 7 days after treatment, and it appeared in the positive control group CK3 group at 10 days. Small plants continued to die over time. In the final group A, the disease index was 16%. In the CK3 group, the disease index was 8%. Although group A did not achieve the same effect as the CK3 group compared with the control, the number of dead plants was greatly reduced, and the disease prevention effect was 78.95% (Fig. 1). After field application comparison, group A showed good yield in the field infected with wilt disease; such yield was similar to the yield of bananas in the completely healthy group CK1. The incidence rate was still higher than CK3 but much lower than CK2. Comprehensive comparison showed that the incidence rate of group A was low, the effect of disease prevention was good, and the yield on the field was excellent (Fig. 2).

### Classic taxonomic features

**Fig. 3:**
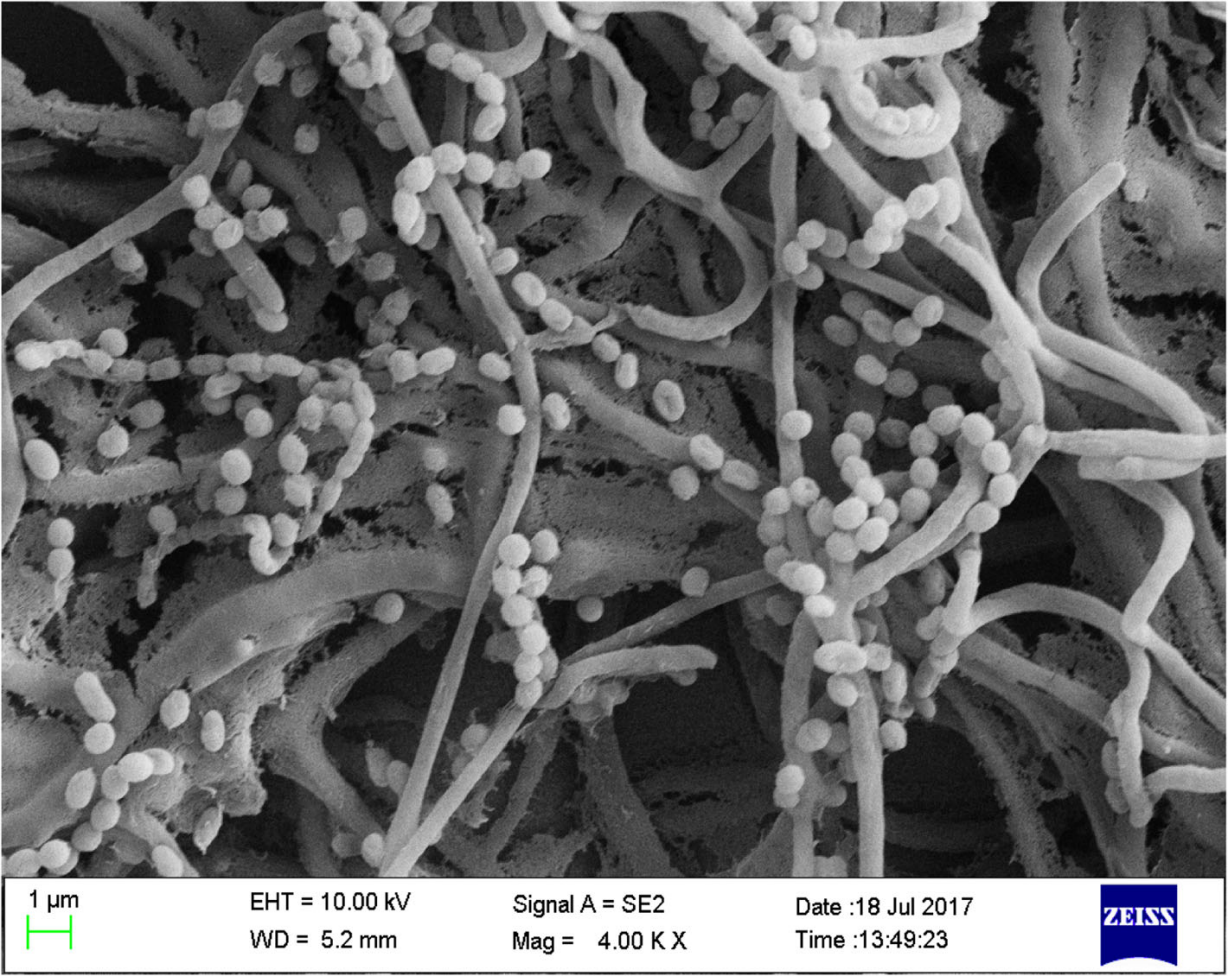
Morphological characteristics of spore chain and spore of strain XL-6 (4.00 KX)

The antagonistic actinomycetes were cultured using the insertion method. The results of transmission electron microscopy showed that the filaments of strain *FS-4* were straight and flexible, and the spores were oval (Fig. 3). The strain *FS-4* grew well on seven kinds of cultures. The colony morphology was shown in Additional file 1: Table S1. The aerial mycelium was mainly white and gray white, and the glycerol asparagine agar (ISP7) culture was bean yellow in color. The mycelium in the base strain was rich in color in different cultures, and no soluble pigment was produced.

The *FS-4* actinomycete can do the following: reduce nitrate; hydrolyze starch; and produce H_2_S, melanin, urease, and tyrosinase. However, it cannot liquefy gelatin and milk peptone and did not exhibit solidification. The pH range of growth was 5.0–10.0, and the optimum pH for growth was 7.0. The optimum growth temperature was 28 °C–32 °C. It did not grow on culture with NaCl content greater than 3%. Other physiological and biochemical characteristics are shown in Additional file 2: Table S2.

### Phylogenetic characteristics

**Fig. 4:**
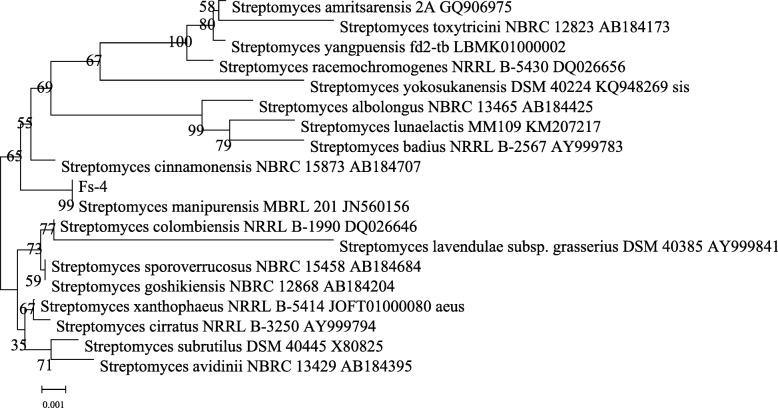
Phylogenetic tree of the *FS-4* strain based on 16S rRNA gene sequences. Note: The bootstrap values (%) presented at the branches were calculated from 1000 replications. The numbers in parentheses are GenBank accession numbers. The scale bar 0.001 represents two nucleotide substitutions per 1000 nucleotides

According to Fig. 4, the strain *FS-4* and *Streptomyces* ma. were clustered in the same branch and had high similarity in sequence with a similarity coefficient of 99%. The target strain was identified as *Streptomyces* ma, according to the following: results of cell morphology; culture; physiological and biochemical characteristics of the strain *FS-4*; and phylogenetic analysis based on the 16 s rRNA.

### Optimization of fermentation conditions on Bacteriostasis of the *Streptomyces* ma*.FS-4*

#### Carbon source and content

**Fig. 5:**
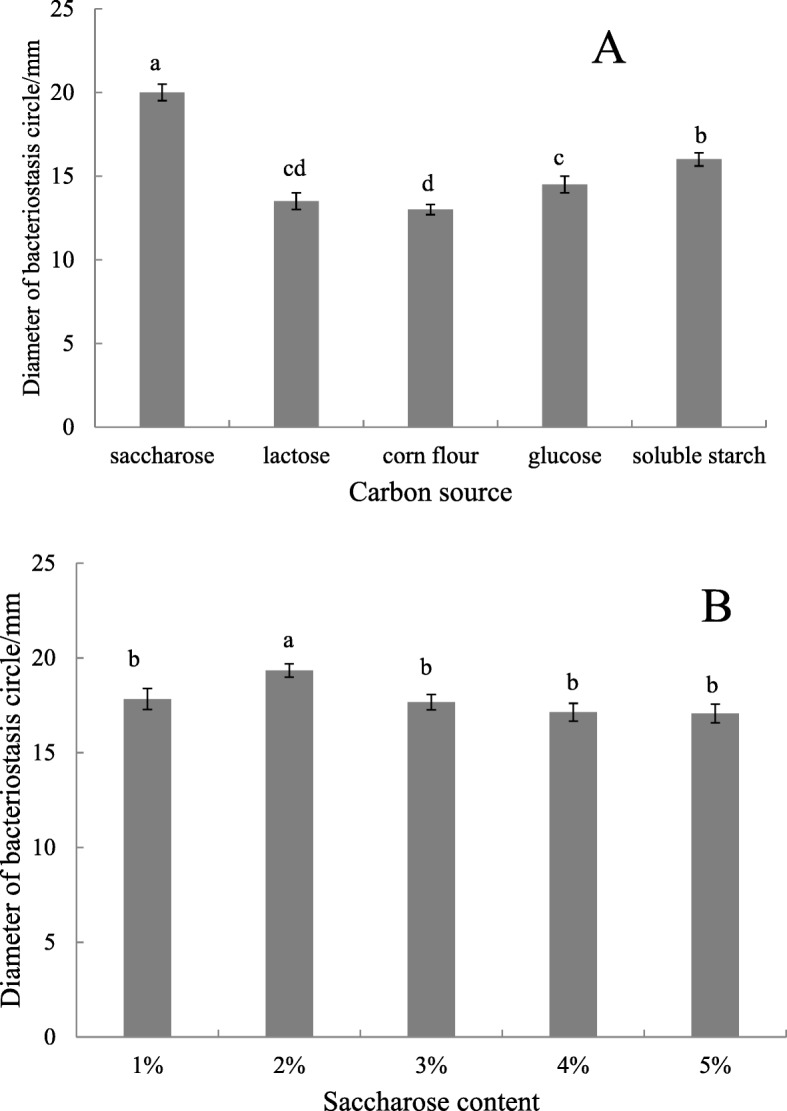
Effect of carbon sources on the activity of antimicrobial substance produced by the F-4 strain. Note: **A** (carbon type), **B** (Saccharose content), Different lowercase letters show significant difference at *P* < 0.05

The carbon source was optimized on the basis of Gause’s no. 1 culture. Saccharose, lactose, corn flour, glucose, and soluble starch were used as carbon sources. As shown in Fig. 5a, the maximum diameter of the inhibition circle of fermentation broth reached 20.5 mm on the culture with sucrose as carbon source. Saccharose was the most favorable carbon source for the *Streptomyces* ma*. FS-4* to produce bacteriostatic substances. By adjusting saccharose at 1–5%, the optimal carbon source concentration was achieved. As shown in Fig. 5b, when the sucrose content was 2%, the fermentation broth had the largest bacteriostasis circle and the highest number of antimicrobial substance.

#### Nitrogen source and content

**Fig. 6:**
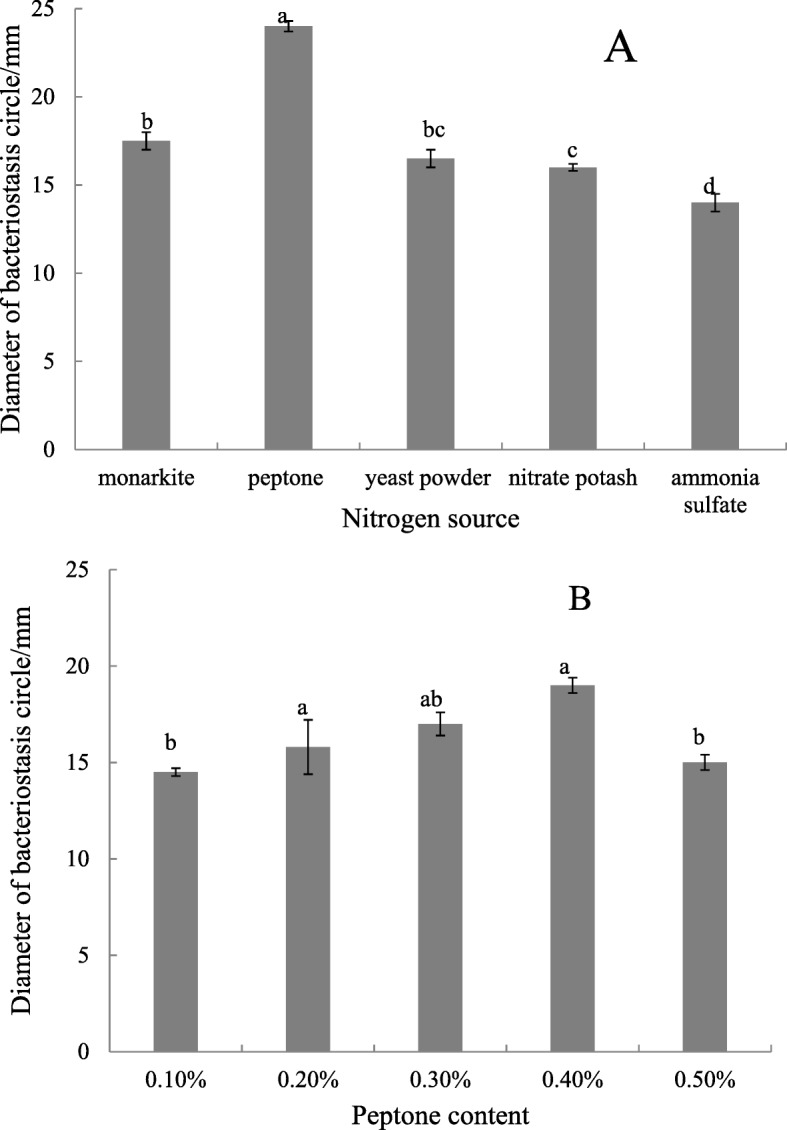
Effect of carbon sources on activity of antimicrobial substance produced by the FS-4 strain. Note: **A** (nitrogen type), **B** (Saccharose content), Different lowercase letters show significant difference at *P* < 0.05

The nitrogen source was optimized on the basis of Gause’s no. 1 culture. As shown in Fig. 6a, the culture with peptone was the best nitrogen source with a diameter of 24 mm. When the peptone content was 0.2%, the fermentation broth had the largest inhibition circle and the highest number of antimicrobial substances (Fig. 6b).

#### Inorganic salt concentration

**Fig. 7:**
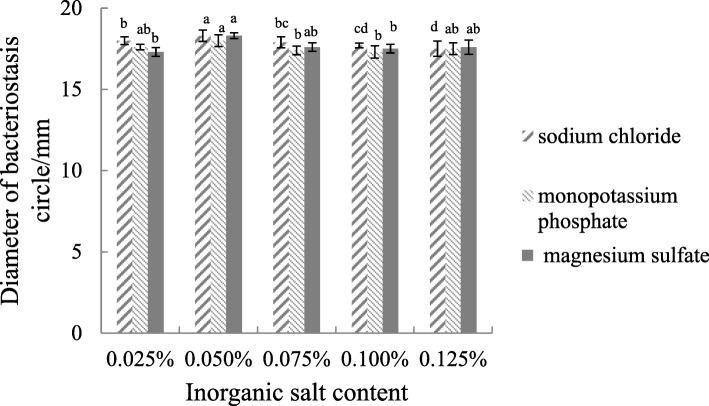
Effect of inorganic salt content on the activity of antimicrobial substance produced by the *FS-4* strain. Note: Different lowercase letters show significant difference at *P* < 0.05

The concentration of inorganic salt was optimized on the basis of Gause’s no. 1 culture, as shown in Fig. 7. The regulation of three kinds of inorganic salt concentrations had little effect on antibacterial substances produced by the *Streptomyces* ma*.FS-4*.

#### Different culture conditions

**Fig. 8:**
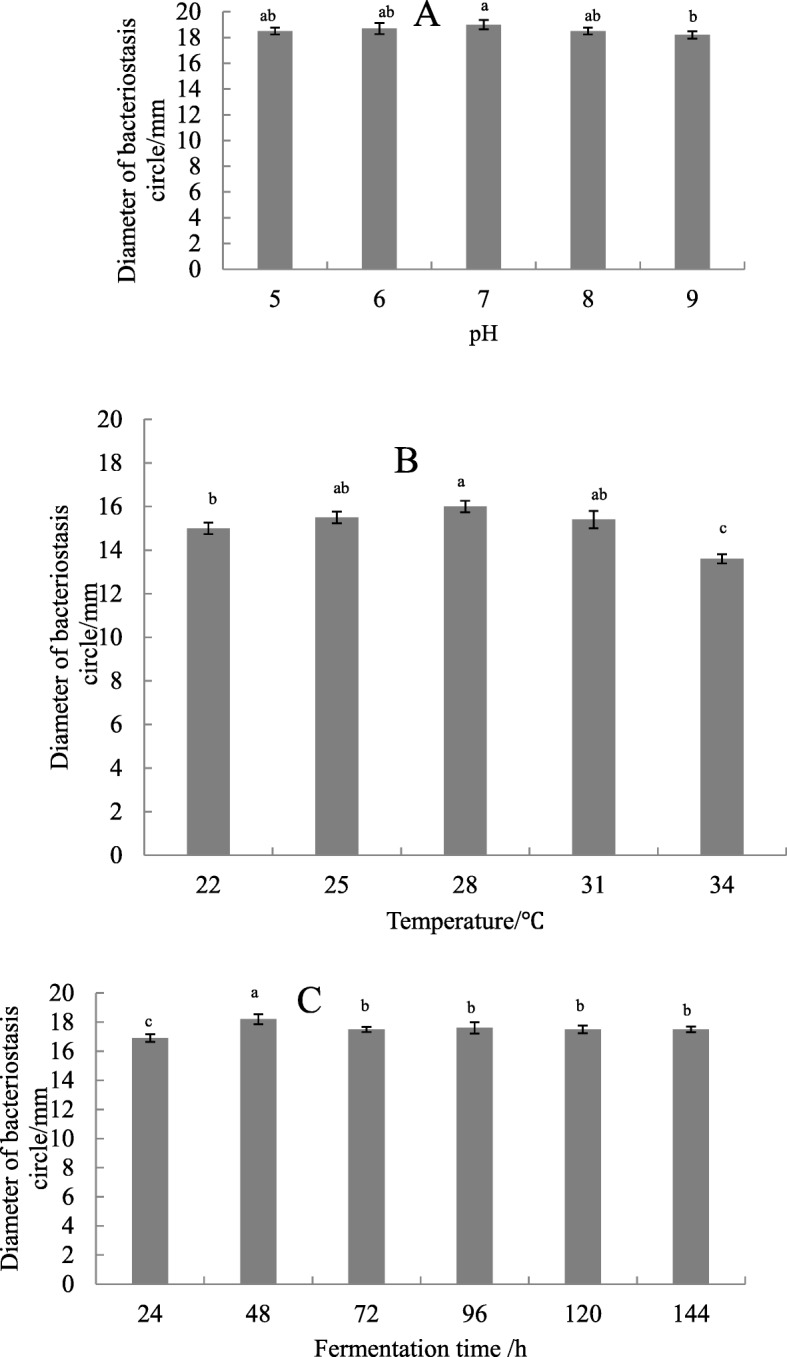
Effect of different conditions on the inhibition activity of the submerged and cultured FS-4. Note: **A** (pH) , **B** (temperature ), **C** (fermentation time), Different lowercase letters show significant difference at *P* < 0.05

Appropriate pH value, fermentation temperature, and time contributed to the production of antimicrobial substances by *Streptomyces* ma*. FS-4*. Figure 8a shows that the diameter of the bacteriostasis circle varied slightly at pH 6 to 8. When the pH was 7, the maximum diameter of the bacteriostasis circle was 19 mm. When the temperature was 22 °C–31 °C, the diameter of the bacteriostasis circle changed slightly. However, when the temperature was 28 °C, the bacteriostasis circle had the largest diameter (Fig. 8b). The diameter of the bacteriostasis circle reflected the number of antimicrobial substances in the fermentation broth for 24 h. The diameter of the bacteriostasis circle no longer increased after 48 h of fermentation. *Streptomyces* ma*. FS-4* basically stopped producing antimicrobial substances or the activity of antimicrobial substances decreased (Fig. 8c).

#### Analysis and optimization of response surface

The results of response surface experiments were fitted with multivariate regression. The quadratic regression equation between the diameter of inhibition circle and peptone concentration, sucrose concentration, and time were as follows: Y = 25.26 + 1.94A + 0.31B + 1.63C – 0.25AB + 0.88 AC + 0.38 BC – 0.88A^2^–0.38B^2^–2.26C^2^. The ANOVA for the regression equation model is presented in Additional file 3: Table S3.

The graph of response surface analysis can intuitively reveal the influence of the variables and their interactions on the response value. The response surface obtained from the regression model is shown in Additional file 5: Figure S1.

Additional file 4: Table S4 showed that the regression equation model was very significant, thereby indicating that the model was reliable. The correction coefficient of the model was 0.9094, thereby showing that the fitting model was good. The influence on the diameter of bacteriostasis was in the following order: peptone > saccharose > time.

The optimum conditions were obtained by Design-Expert V8.0.6 software: 0.5% peptone, 2.37% sucrose, and time 62.04 h. Under this condition, the theoretical value of the bacteriostasis circle diameter was 27.06 mm. To facilitate the experimental operation, the conditions were revised as follows: peptone concentration 0.5%, sucrose concentration 2.4%, and time 62 h. The diameter of the bacteriostasis circle was 26.7 mm, which was close to the predicted theoretical value.

## Discussion

*Fusarium* wilt of banana is a devastating disease in banana planting. From the viewpoint of soil microbial diversity, the growth and reproduction of pathogenic fungi can be effectively controlled only by increasing the diversity of antagonistic microorganisms in soil [5, 6, 9]. Actinomycetes are abundant bioactive material resources. They can produce antibacterial substances, degrade the cell wall and enzymes of fungi, and are a kind of microbial population with great practical value [19, 24, 25]. In this study, stable and antagonistic actinomycetes were isolated from banana rhizosphere soils that infested with severe *Fusarium* wilt disease. The strain was named *Streptomyces* ma*. FS-4*. Biological organic fertilizer was made from the fermentation broth of actinomycetes *FS-4* and used for the biological control of banana wilt. The disease was controlled from the point of view of micro-ecology. Actinomycete *FS-4* fermentation broth showed good control effect on infected banana seedlings, thereby reducing their disease index and improving the prevention and control effects of banana plant wilt. Compared with the control, the prevention and control effects of actinomycete *FS-4* fermentation broth was significant; the disease prevention effect reached 78.95%, and the disease index was only 16%. Thangavelu et al. [30] isolated *Bacillus subtilis* from banana root soil, and the control effect of Fusarium wilt of banana can reach 41%. Nel et al. [31] screened out strains *CAV255* and *CAV241* from anti-bacterial bacteria, whose disease-prevention capacities for Fusarium wilt of banana were 87.4 and 75%, respectively. Therefore, actinomycete *FS-4* fermentation broth can be used in production and field protection.

The spores of strain *FS-4* were oval, compared with *S. manipurensis* PH2494 [21], *Streptomyces spectabilis* PH2792 [24], and *S. spectabilis* B4491 [14]. All of these species can hydrolyze starch and produce H_2_S ad brown pigment. However, PH2792 and PH2494 can liquefy gelatin and reduce nitrate, but B4491 cannot. Moreover, strain *FS-4* can reduce nitrate but cannot liquefy gelatin. In terms of carbon usage, all can utilize glucose, fructose, mannitol, galactose, and soluble starch. However, PH2792 cannot use sucrose; PH2494 cannot use sucrose; B4491 cannot use arabinose, xylose, inositol, chrysanthemum, and sorbitol; and strain *FS-4* cannot use sucrose, trehalose, and inositol. In terms of culture characteristics, strain *FS-4* was highly similar to the other three *Streptomyces* strains. The main hyphae were white and grey white, and the inner hyphae were orange powder to orange red. The difference between strain *FS-4* and the three other *Streptomyces* strains was that strain *FS-4* had a root-like inner hyphae, and its main hyphae consisted of a polybranch. The spore silk of strain *FS-4* was also long, straight, and gently curved. Strain *FS-4* was further identified as *S. manipurensis*.

*S. manipurensis* can produce various antibiotics with high bacteriostatic activity, thereby showing its high application value in medicine. However, few researchers have studied the biological control effect of *S. manipurensis* on plant diseases [19, 21]. Through the study of antagonistic actinomycete *FS-4* broad-spectrum resistance, researchers found that this strain exerted a good inhibitory effect on soil-borne pathogens, such as *F. oxysporum* f. sp. *cubense* and *A. tenuissima* [14, 17]. However, field and plant microecological environment influence the bacteriostatic effect of actinomycete *FS-4* [19, 22]. Accordingly, to improve the use of actinomycete *FS-4* in actual production, follow-up studies on the separation and identification of antiseptic active substances in the fermentation solution are required. Such studies should explore the mechanism of antiseptic bacteria and field-protective effects. Antifungal-compound production also has the crucial effect of inhibiting soilborne fungal pathogens. Ling et al. [32] reported that interactions between beneficial microbe and the fungal pathogen have evolved to enable efficient antibiotic production and survival in the environment.

Antimicrobial active substance is typically used as the base on which antagonistic actinomycetes exert biological effect. The yield of antimicrobial active substance determines the actual effect of bacteriostasis [13, 20, 25]. Fermentation is the basis for obtaining numerous microbial active metabolites. The type and yield of microbial metabolites are closely related to their culture conditions, such as carbon source in culture and pH value of nitrogen source [19, 21, 24]. Carbon source is the energy source for microbial metabolism, and the selection of suitable carbon source can increase the rate of microbial reproduction and metabolite production [14, 15]. Nitrogen is an important element for nucleic acid and protein, which are the raw materials synthesized by microorganisms to create cellular metabolites [15, 21, 25]. The pH affects the ability of bacteria to absorb nutrients and produce active substances. Fermentation temperature affects the growth of microorganisms and the production of secondary metabolites; time affects the production and activity of secondary metabolites [18, 20]. Therefore, exploring suitable culture conditions is important. In this study, the fermentation conditions of *Streptomyces* ma*. FS-4* were optimized by single factor and response surface test. The nutrient base formula and culture conditions of fermentation were obtained; these were suitable for the production of antimicrobial active substances by the target strains. The bacteriostatic activity of the optimized fermentation broth was significantly higher (*P* < 0.05) than that of the basic fermentation culture. The maximum bacterial ring diameter of optimized fermentation solution for *Bacillus subtilis* was 26.7 mm, whereas that of the basic fermentation culture was 23.7 mm, which was an increase of 12.65%.

Microbial fermentation is a dynamic biological process. The pH, oxygen capacity, and product quantity of various nutrient contents in the culture are constantly changed, and various different factors exert a great effect on the types and yield of fermentation products [15, 17, 20]. In this study, only the state of culture and culture conditions before fermentation were considered, and the factors of fermentation process were not monitored dynamically.

## Conclusions

Actinomycete *FS-4* could serve as a biological control to inhibit five pathogenic fungi and promote banana growth. Pot experiments showed that the incidence of banana seedlings was reduced after using *Streptomyces* ma*. FS-4* treatment. The optimum fermentation conditions were tested, and the diameter of the inhibition circle was found to be 26.7 mm. Combined with morphological observation, physiological and biochemical determination, and the phylogenetic method, strain *FS-4* was identified as *Streptomyces* ma.

## Supplementary information

**Additional file 1: Table S1.** Medium characteristics of strain *FS-4.*

**Additional file 2: Table S2.** Physiological and biochemical characteristics of strain *FS-4.*

**Additional file 3: Table S3.** Results of response surface design experiments.

**Additional file 4: Table S4.** ANOVA for regression equation model.

**Additional file 5: Figure S1.** Effect of various factors on activity of antimicrobial substance produced by *FS-4.*

## Data Availability

All data generated or analyzed in this study are presented within this manuscript. All materials used in this study including raw data shall be available upon reasonable request.

## Abbreviations

ma: manipurensis
PDA: Potato Dextrose Agar
