# Supplementary material for: Fermentation optimization and disease suppression ability of a Streptomyces ma. FS-4 from banana rhizosphere soil
Source: BMC Microbiol. 2020 Jan 31;20:24. doi: 10.1186/s12866-019-1688-z (PMC6995205; doi:10.1186/s12866-019-1688-z)
Supplement: Supplementary file 1 — Additional file 1: Table S1. Medium characteristics of strain FS-4. [file 12866_2019_1688_MOESM1_ESM.docx]

**Table S1.** Medium characteristics of strain *FS-4*

| **Medium** | **Growth** | **Bacterial colony** | **Aerial mycelium** | **Substrate mycelium** | **Soluble pigment** |
| --- | --- | --- | --- | --- | --- |
| ISP1 | good | gray | hoar | tan | none |
| ISP2 | good | hoar | hoar | deep yellow | none |
| ISP3 | good | bright brown | white | buff | none |
| ISP4 | good | white | white | ivory | none |
| ISP5 | good | ivory | none | ivory | none |
| ISP6 | good | celadon | none | taupe | none |
| ISP7 | good | gray | bean yellow | light bean yellow | none |
