# Supplementary material for: Fermentation optimization and disease suppression ability of a Streptomyces ma. FS-4 from banana rhizosphere soil
Source: BMC Microbiol. 2020 Jan 31;20:24. doi: 10.1186/s12866-019-1688-z (PMC6995205; doi:10.1186/s12866-019-1688-z)
Supplement: Supplementary file 2 — Additional file 2: Table S2. Physiological and biochemical characteristics of strain FS-4. [file 12866_2019_1688_MOESM2_ESM.docx]

**Table S2.** Physiological and biochemical characteristics of strain *FS-4*

| **Characteristics** | **Results** | **Characteristics** | **Results** |
| --- | --- | --- | --- |
| Biochemical test |  | Sugar utilization |  |
| Gelatin liquefaction | + | α-Lactose | - |
| Pigment | + | D-Cellobiose | + |
| CAT | + | D-Fructose | + |
| Nitrate reduction | + | D-Galactose | + |
| Amylolysis | + | D-Glucose | + |
| MR | + | D-Mannose | + |
| TYR | + | D-Sorbitol | + |
| H_2_S | - | D-Trehalose | - |
| Nitrogen utilization |  | D-Xylose | + |
| α-Naphthalene acid | - | L-Arabinose | + |
| L-Arginine | + | L-Phenylalanine | + |
| L-Serine | + | Melitose | - |
| L-Phenylalanine | + | Melibiose | + |
| Glycine | + | Xylan | + |
| Methionine | + | D-Mannitol | - |
| L-Hydroxyproline | - | Inositol | + |
| L(+)-Cysteine | - | Melezitose | + |
| L-Homocysteine | + | Rhamnose | + |
| Valine | + | Ribose | - |
| Histidine | + | Saligenin | + |
| Ammonium nitrate | + | Soluble starch | + |
| Ammonium chloride | - | Sucrose | + |

**Note**: “+”: positive; “-”: negative.
