# Supplementary material for: Fermentation optimization and disease suppression ability of a Streptomyces ma. FS-4 from banana rhizosphere soil
Source: BMC Microbiol. 2020 Jan 31;20:24. doi: 10.1186/s12866-019-1688-z (PMC6995205; doi:10.1186/s12866-019-1688-z)
Supplement: Supplementary file 3 — Additional file 3: Table S3. Results of response surface design experiments. [file 12866_2019_1688_MOESM3_ESM.docx]

**Table S3.** Results of response surface design experiment**s**

| Experiment number | A  peptone (%) | B  sucrose (%) | C  fermentation time (h) | Diameter of bacteriostasis circle |
| --- | --- | --- | --- | --- |
| 1 | 0.4 | 2 | 48 | 25 |
| 2 | 0.5 | 2 | 24 | 21.5 |
| 3 | 0.4 | 3 | 24 | 20.5 |
| 4 | 0.3 | 1 | 48 | 21 |
| 5 | 0.4 | 2 | 48 | 24.5 |
| 6 | 0.3 | 3 | 48 | 22.5 |
| 7 | 0.3 | 2 | 72 | 21 |
| 8 | 0.4 | 2 | 48 | 25 |
| 9 | 0.5 | 1 | 48 | 26 |
| 10 | 0.4 | 2 | 48 | 25.5 |
| 11 | 0.4 | 3 | 72 | 25 |
| 12 | 0.5 | 2 | 72 | 26 |
| 13 | 0.4 | 1 | 72 | 24 |
| 14 | 0.5 | 3 | 48 | 26.5 |
| 15 | 0.4 | 1 | 24 | 21 |
| 16 | 0.3 | 2 | 24 | 20 |
| 17 | 0.4 | 2 | 48 | 26.3 |
