# Supplementary material for: Fermentation optimization and disease suppression ability of a Streptomyces ma. FS-4 from banana rhizosphere soil
Source: BMC Microbiol. 2020 Jan 31;20:24. doi: 10.1186/s12866-019-1688-z (PMC6995205; doi:10.1186/s12866-019-1688-z)
Supplement: Supplementary file 4 — Additional file 4: Table S4. ANOVA for regression equation model. [file 12866_2019_1688_MOESM4_ESM.docx]

**Table S4.** ANOVA for regression equation model

| Variance source | Quadratic sum | Free degree | Mean square | *F* value | *p* | Significance |
| --- | --- | --- | --- | --- | --- | --- |
| Model | 82.73 | 9 | 9.19 | 18.85 | 0.0004 | ** |
| A Peptone | 30.03 | 1 | 30.03 | 61.57 | 0.0001 | ** |
| B Saccharose | 0.78 | 1 | 0.78 | 1.60 | 0.2462 |  |
| C Time | 21.13 | 1 | 21.13 | 43.31 | 0.0003 | ** |
| AB | 0.25 | 1 | 0.25 | 0.51 | 0.4972 |  |
| AC | 3.06 | 1 | 3.06 | 6.28 | 0.0407 | * |
| BC | 0.56 | 1 | 0.56 | 1.15 | 0.3185 |  |
| A^2^ | 3.26 | 1 | 3.26 | 6.68 | 0.0362 | * |
| B^2^ | 0.61 | 1 | 0.61 | 1.25 | 0.3011 |  |
| C^2^ | 21.41 | 1 | 21.41 | 43.89 | 0.0003 | ** |
| Residual | 3.41 | 7 | 0.49 |  |  |  |
| Lost term | 1.56 | 3 | 0.52 | 1.12 | 0.4386 |  |
| Pure error | 1.85 | 4 | 0.46 |  |  |  |
| Sum | 86.15 | 16 |  |  |  |  |
