# Supplementary figures and images for: Fermentation optimization and disease suppression ability of a Streptomyces ma. FS-4 from banana rhizosphere soil
Source: BMC Microbiol. 2020 Jan 31;20:24. doi: 10.1186/s12866-019-1688-z (PMC6995205; doi:10.1186/s12866-019-1688-z)

**Figuure S1**


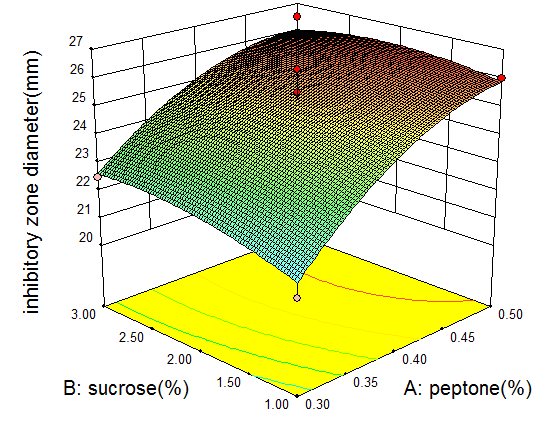


**
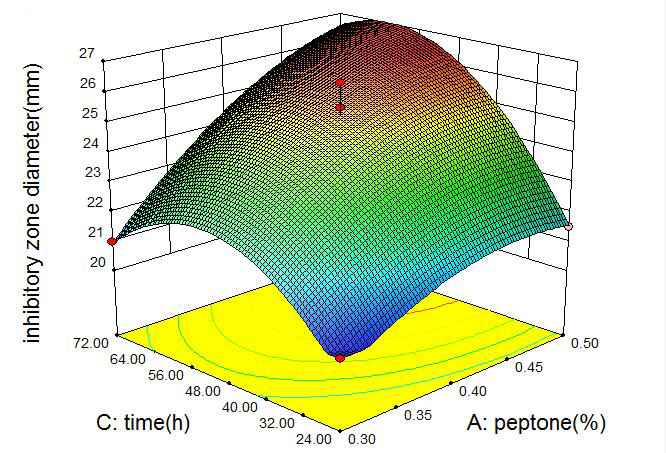
**


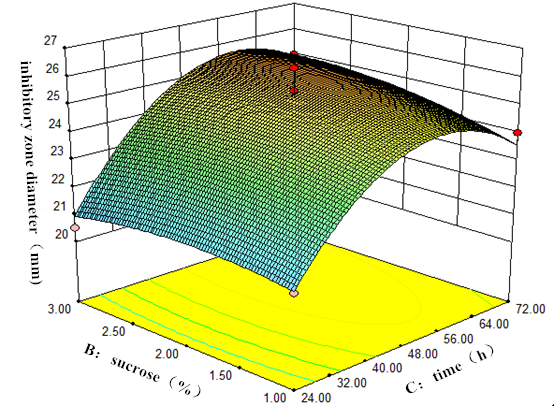

Supplement: Supplementary file 5 — Additional file 5: Figure S1. Effect of various factors on activity of antimicrobial substance produced by FS-4. [file 12866_2019_1688_MOESM5_ESM.docx]
